# BioPP: a tool for web-publication of biological networks

**DOI:** 10.1186/1471-2105-8-168

**Published:** 2007-05-22

**Authors:** Ganesh A Viswanathan, German Nudelman, Sonali Patil, Stuart C Sealfon

**Affiliations:** 1 Center for Translational Systems Biology and Department of Neurology, Mount Sinai School of Medicine, New York, NY 10029, USA

## Abstract

**Background:**

Cellular processes depend on the function of intracellular molecular networks. The curation of the literature relevant to specific biological pathways is important for many theoretical and experimental research teams and communities. No current tool supports web publication or hosting of user-developed large scale annotated pathway diagrams. Sharing via web publication is needed to allow real-time access to the current literature pathway knowledgebase, both privately within a research team or publicly among the outside research community. Web publication also facilitates team and/or community input into the curation process while allowing centralized control of the curation and validation process. We have developed new tool to address these needs. Biological Pathway Publisher (BioPP) is a software suite for converting CellDesigner Systems Biology Markup Language (CD-SBML) formatted pathways into a web viewable format. The BioPP suite is available for private use and for depositing knowledgebases into a newly created public repository.

**Results:**

BioPP suite is a web-based application that allows pathway knowledgebases stored in CD-SBML to be web published with an easily navigated user interface. The BioPP suite consists of four interrelated elements: a pathway publisher, an upload web-interface, a pathway repository for user-deposited knowledgebases and a pathway navigator. Users have the option to convert their CD-SBML files to HTML for restricted use or to allow their knowledgebase to be web-accessible to the scientific community. All entities in all knowledgebases in the repository are linked to public database entries as well as to a newly created public wiki which provides a discussion forum.

**Conclusion:**

BioPP tools and the public repository facilitate sharing of pathway knowledgebases and interactive curation for research teams and scientific communities. BioPP suite is accessible at

## Background

Understanding cellular function requires detailed cell-type specific insight into the structure and operation of the molecular networks formed by a cell's genes and proteins [1]. The communication among cellular components is governed by logical and signal transfer processes [2]. These interactions are typically represented as a directed wiring diagram showing aspects of signaling, metabolic and gene pathway connections [3,4]. Cellular pathway maps provide insight into network topology and capture the relationship of the nodes, and into the underlying biological responses. Due to the complexity of these networks, there is increasing impetus to create utility-based software tools to construct, visualize, and analyze pathways [5].

**Figure 1:**
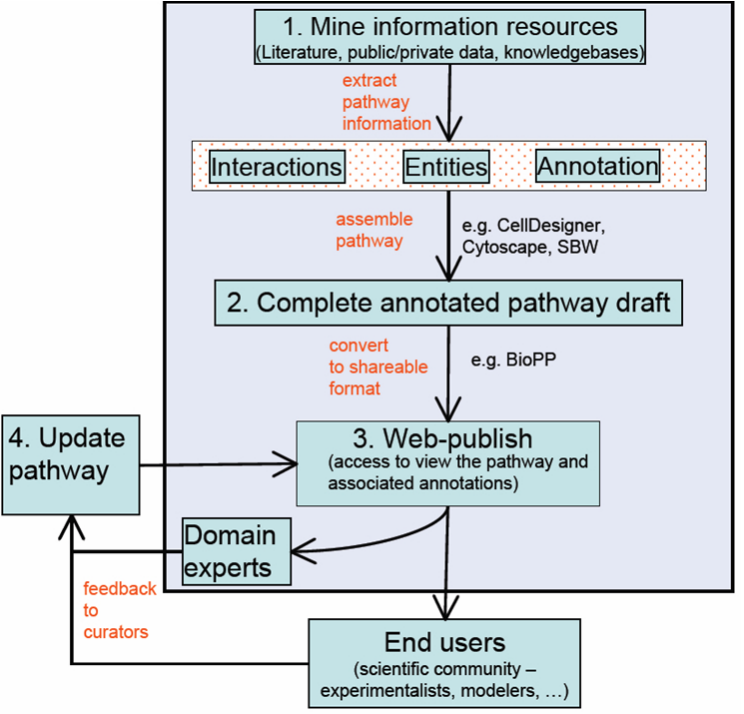
Schematic illustrating the process of interative manual curation of a biological pathway. Pathway curators initially mine information sources and assemble a pathway draft. Web publication of the resulting annotated pathway can facilitate user access to the information. In addition, web publication facilitates community and domain expert feedback leading to correction, refinement and improvement of the pathway knowledgebase content and user access to the current release.

Biological network curation involves the construction, verification and refinement of an annotated pathway map. Maps may be generic or cell-type specific. Accurate assembly of an annotated pathway map is a difficult, slow, iterative and labor-intensive project (Fig 1). Curators use various databases and text-mining tools to identify relevant citations or data [6]. Curation often involves coordinating input from a large group of scientists including biological curators, domain experts, collaborators and the scientific community. The high granularity of a detailed network makes visualization and exploration of the underlying knowledgebase difficult. Improving accessibility to the network map and annotations is important both for facilitating the iterative curation process and for sharing the most current knowledgebase within research teams and with the scientific community.

Most pathway maps are created using standalone software tool and stored in modifications of the Systems Biology Markup Language (SBML) [7-9]. The complexity of biological pathways can lead to the construction of breathtakingly detailed wiring diagrams which are very difficult to use and verify [10]. Network maps published in the literature are fixed in time, do not provide flexible access to the detailed underlying knowledgebase, and are difficult to read [11,12].

There are several software tools that provide good drawing platforms to build, annotate and visualize biological pathways. Tools that provide automated layout of SBML files have been developed as part of the Systems Biology Workbench [13,14]. Cytoscape is another excellent open source software environment for the construction of biological networks [15]. We have found that limitations in the symbolic representation of nodes, in drawing and layout tools and in visual representation using the current releases of Systems Biology Workbench SBML layout extension or Cytoscape make it difficult to construct an easily understood diagram for high granularity cell-specific signaling maps. For detailed networks, we find that CellDesigner [16] provides functional graphic tools, pathway visualization and navigation. However, CellDesigner is a standalone desktop application. As network maps rapidly evolve, this type of implementation makes the real-time sharing of the knowledgebase difficult.

In order to facilitate the incorporation of new information and the correction of errors in a detailed pathway map, web-based dissemination of the map and knowledgebase is required. Current web-based pathway maps, such as PANTHER [17] and Reactome [18,19], do not provide publicly accessible tools for the conversion of user-created pathways into a web-accessible form. We were motivated to develop a pathway publishing tool for CD-SBML files in order to meet the present needs of our curation team. Our experience with the desktop application BioPathwise (BioAnalytics Group LLC), which integrates pathway drawing and web publication of small scale networks, indicated the importance of such a tool for the curation and dissemination of large scale maps constructed in CellDesigner. We believe this will be useful for many research teams that are constructing specialized pathway maps. In developing this tool, we also incorporated several helpful features not present in CellDesigner that assist access to the annotations. In order to fill the need for a mechanism to share specialized pathway maps developed by different groups, we have also established a pathway repository that automatically web-publishes user-created CellDesigner-based maps and knowledgebases.

## Implementation

The BioPP suite consists of four interrelated elements: a pathway publisher, an upload web-interface, a pathway repository and a pathway navigator, each of which is described separately below. The pathway publisher uses information provided through the pathway upload web-interface to web-publish the pathway and/or to populate the pathway repository. The networks in the pathway repository are publicly viewable via the pathway navigator. The BioPP suite is community accessible (see Availability and requirements).

### Pathway publisher

This Perl implemented *publisher *application is launched on a dedicated server that hosts the BioPP suite through the pathway *upload web-interface*. The pathway publisher receives the user-uploaded information about the pathway, and the associated CD-SBML file and the png image exported from CellDesigner 4.0(alpha). The publisher parses the uploaded files to create a flat html file library describing the underlying pathway content and mapping the entity coordinates in the pathway. The hyperlinks on the entities point to the corresponding annotation information. The library contains various types of information such as sorted lists of different types of entities (Protein/Gene/RNA), sorted list of interactions, annotation for each interaction and for each entity, and the co-ordinates of entities.

The user can also choose to have the network published, using an automated procedure, into the pathway repository. After publication, the user will be returned (via browser) the converted files, in standard compressed zip format. Should the user prefer not to publish the converted pathway in the public pathway repository, the converted files are returned back to the user, and all user-uploaded information and converted files are deleted from the server. Thus, if desired, users can utilize the publisher to convert pathways for their own use without compromising confidential information.

### Upload web-interface

**Figure 2:**
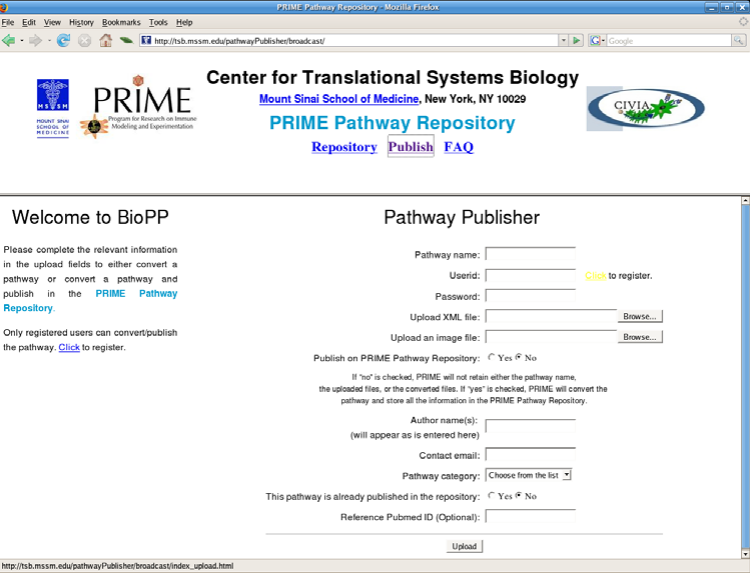
The upload interface of the BioPP Suite.

The upload web-interface is based on CGI and is implemented in Perl. Following a simple registration process, a user receives an e-mail with a username and corresponding password, which will enable the user to upload the biological network related information, such as pathway name, CD-SBML file, .png image file, and other identifying information onto the server via the web-interface (Fig. 2). The uploaded user details, the network's information and the uploaded files are validated both on the client side, using javascript, and on the server side. Upon successful network content verification and upload, the pathway publisher is launched on the server side for conversion of the network and, if requested by the user, publication in the pathway repository. The converted files, in standard compressed zip format, are then presented to the user for download.

### Pathway repository

**Figure 3:**
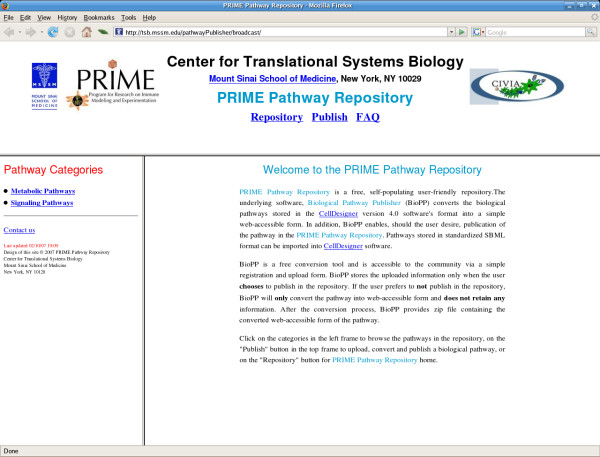
The web-accessible pathway repository.

The pathway *repository *(Fig. 3) is a flat-file based database containing user-uploaded pathways. Pathways are converted into flat-files and/or published in the pathway repository by the pathway *publisher*. A previously published pathway can be unpublished by the author by emailing the repository administrator. We have also implemented a mechanism to deal with updating pathways and with modification of existing pathways generating new, modified pathways. The repository provides version control, with old and new versions of the pathways remaining accessible. User registration is not required to view and navigate any of the pathways that are deposited in the public repository.

### Pathway navigator

**Figure 4:**
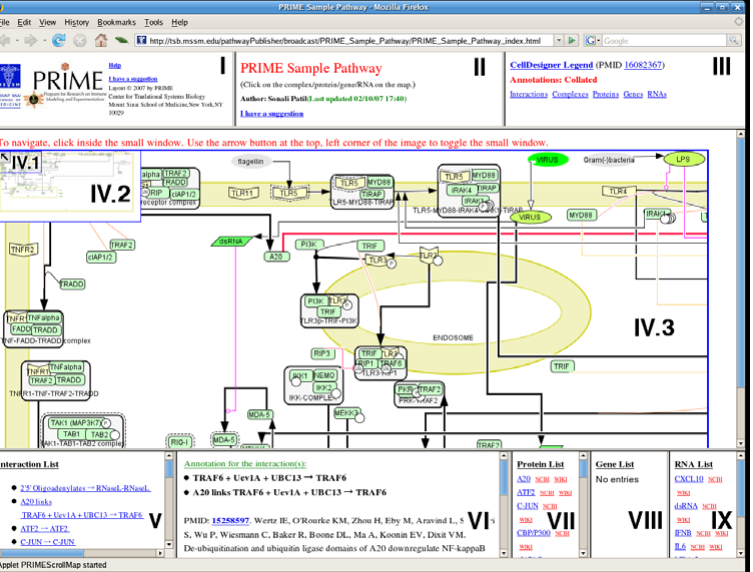
The web-accessible pathway navigator. A sample pathway is shown that represents part of the viral activated signaling pathways in human dendritic immune cells. The entities and links shown in the pathway are clickable to display the corresponding annotations. The content of the individual frames, which are indicated by roman numerals, are as follows: I – Help link. II – brief author provided description of the pathway and contact information for suggesting pathway revisions. III – link for the pathway legend (from CellDesigner), and links for collated lists of the annotations for interactions, protein, genes, and RNAs in the network. IV.1 navigating zoom rectangle. Dragging the zoom rectangle or clicking inside the index window (IV.2) displays the appropriate section of the pathway in IV.3. The arrow in IV.1 is a toggle button to hide/unhide the index window. The zoom facilitates navigating, particularly when the network map is large and fine-grain. V, VII, VIII, IX – sorted, hyperlinked lists of all the interactions, proteins, genes and RNAs, respectively. Clicking on a hyperlink displays the annotations in the annotation frame (VI). Links to the Entrez gene pages and the appropriate page of a public wiki-based discussion forum also launch new windows. VI – annotation frame which displays selected annotations. Clicking an interaction displays the annotations. Clicking a PMID opens a separate PubMed window for that citation.

The pathway *navigator *was developed to facilitate browsing the uploaded pathway (Fig. 4). The navigator is implemented as a multi-frame HTML web page, each frame of which presents a network specific information, such as the network, list of interactions, annotations and lists of various entities. In order to facilitate easy exploration of large networks, the navigator is enabled with a JAVA applet based panning facility to pan the network via a zoom rectangle in an index window containing the thumbnail of the original image.

## Results and discussion

The BioPP suite provides a unique presentation of the biological pathways that facilitates sharing relevant information with the research team or the scientific community. BioPP converts the annotated pathways stored in CellDesigner's SBML format (CD-SBML) into a HTML library used for web-publication of the pathways. As CellDesigner permits import and conversion of the standard SBML format into CD-SBML, BioPP can be used to convert any pathway stored in the standard SBML format. Therefore, BioPP permits a very easy, flexible and quick access to large amounts of biological information. The pathways are presented in several HTML frames (Fig. 4), each with different types of useful information. We define the primary frame as the *network *frame and the other frames as the introduction, legend, interactions, proteins, genes, RNAs frame (Fig. 4).

The network frame contains a click-enabled image of the biological network. The entities and interactions with supporting annotation, pathway graphics legend and other pertinent information assist exploration of the network. The network is displayed through a JAVA applet which enables panning of the network via the index window, containing a smaller image of the map. Dragging a zoom rectangle inside the index window will result in displaying the underlying map location in the main frame.

All entities in the main frame are hyperlinked to the corresponding annotations, if available. All annotations are presented in the annotation frame. These annotations display a list of interactions in which the chosen entity is involved and the annotation corresponding that entity, if any. Each interaction in this list is hyperlinked to the corresponding interaction annotation, if available. In the introduction and legend frames, we present a brief user-specified description of the network and the CellDesigner legend [20] of the representations in the network.

Experience suggests that the proteins, genes, and interactions involved in an existing pathway are valuable information for the curators and accelerates construction of a pathway. The interactions, proteins, genes, and RNA frames are populated with the sorted list of interactions, proteins, genes and RNAs, respectively. All the interactions that contain relevant annotation are hyperlinked, which when clicked will display the corresponding annotations in the annotation frame. Note that several interactions may be annotated with the same information, e.g. citations. In order to avoid redundancy, the tool ensures that all the annotation HTML files are unique. As a result, should need arise, several interactions are hyperlinked to the same annotation information. In addition, a list of interactions that is annotated by the currently displayed information is presented in the annotation frame. Similarly, all the proteins, genes, and RNAs in the respective frames are hyperlinked.

An entity specific link to NCBI's Gene Entrez page which opens a new window is provided for all the proteins, genes, and RNAs in all the locations wherever cited. In addition, a link to a master, public entity-based wiki page is provided as a forum for community input and discussion.

A completely automated database of biological pathways (with hyperlinks to pertinent annotations) is publicly available (see Availability and requirements). The current interface supports (a) conversion of the original pathway into a web-publishable form and (b) web publication of the pathway in our database. In both cases, the software requires the user to upload the source CD-SBML file and the associated image. Unless the user specifically directs BioPP to publish the pathway to the repository, the server retains no information from the user about the pathway or the HTML pages that are delivered.

## Conclusion

The BioPP suite provides easy to use tools for web-publication and viewing of pathways as well establishes a new public annotated pathway repository. These tools and repository should be useful to pathway curators, to research teams constructing specialized and cell-type specific pathways and to the general research community.

## Availability and requirements

Project Name: Biological Pathway Publisher

Project home page: 

Operating systems: platform independent

Other requirements: Browser supporting Java Virtual Machine (JVM). For viewing large pathways, additional memory allocation to JVM may be required (for instructions on memory allocation see )

Restrictions: none

## Authors' contributions

GAV and GN designed and implemented the software and drafted the manuscript. GAV and SCS conceived of the project. SP tested pathways and helped specify the suite. SCS supervised the project and revised the manuscript. All authors read and approved the final manuscript.
